# Efficacy and safety of intramuscular administration of allogeneic adipose tissue derived and expanded mesenchymal stromal cells in diabetic patients with critical limb ischemia with no possibility of revascularization: study protocol for a randomized controlled double-blind phase II clinical trial (The NOMA Trial)

**DOI:** 10.1186/s13063-021-05430-2

**Published:** 2021-09-06

**Authors:** Barbara Soria-Juan, Mariano Garcia-Arranz, Lucía Llanos Jiménez, César Aparicio, Alejandro Gonzalez, Ignacio Mahillo Fernandez, Luis Riera del Moral, Lukasz Grochowicz, Enrique J. Andreu, Pedro Marin, Gregorio Castellanos, Jose Maria Moraleda, Ana Maria García-Hernández, Francisco S. Lozano, Fermin Sanchez-Guijo, Eva María Villarón, Miriam Lopez Parra, Rosa María Yañez, Antonio de la Cuesta Diaz, Juan Rigoberto Tejedo, Francisco J. Bedoya, Franz Martin, Manuel Miralles, Lourdes del Rio Sola, María Eugenia Fernández-Santos, José Manuel Ligero, Francisco Morant, Luis Hernández-Blasco, Etelvina Andreu, Abdelkrim Hmadcha, Damian Garcia-Olmo, Bernat Soria

**Affiliations:** 1 Jimenez Diaz Foundation University Hospital, FJD Health Research Institute, IIS-FJD UAM, Madrid, Spain; 2 La Paz University Hospital, Madrid, Spain; 3 Navarra University Clinic, Pamplona, Navarra Spain; 4 Virgen de la Arrixaca University Hospital, Murcia, Spain; 5 IBSAL-University Hospital of Salamanca, University of Salamanca, Salamanca, Spain; 6 Hematopoietic Innovative Therapies Division, Centro de Investigaciones Energéticas, Medioambientales y Tecnológicas (CIEMAT), Madrid, Spain; 7 Queen Victoria Eugenia-Cruz Roja Hospital, Sevilla, Spain; 8 University of Pablo de Olavide, Sevilla, Spain; 9 Network Center for Research in Diabetes and Associated Metabolic Diseases (Centro de Investigación Biomédica en Red de Diabetes y Enfermedades Metabólicas Asociadas—CIBERDEM), Instituto de Salud Carlos III, Madrid, Spain; 10 La Fe University Hospital, Valencia, Spain; 11 Valladolid Clinical University Hospital, Valladolid, Spain; 12 Institute for Health Research Gregorio Marañón (IISGM), General University Gregorio Marañón Hospital, Madrid, Spain; 13 Institute for Health Research-ISABIAL, General University Hospital, Alicante, Spain; 14 University Miguel Hernández de Elche, Alicante, Spain; 15 The Spanish Biomedical Research Centre in Diabetes and Associated Metabolic Disorders (CIBERDEM), Madrid, Spain; 16 University of Alicante, Alicante, Spain

**Keywords:** Phase II clinical trial, Randomized, Adipose-derived mesenchymal stromal cells, Critical limb ischemia, Diabetes mellitus, Cell therapy, Advanced therapy medicinal products

## Abstract

**Background:**

Chronic lower limb ischemia develops earlier and more frequently in patients with type 2 diabetes mellitus. Diabetes remains the main cause of lower-extremity non-traumatic amputations. Current medical treatment, based on antiplatelet therapy and statins, has demonstrated deficient improvement of the disease. In recent years, research has shown that it is possible to improve tissue perfusion through therapeutic angiogenesis. Both in animal models and humans, it has been shown that cell therapy can induce therapeutic angiogenesis, making mesenchymal stromal cell-based therapy one of the most promising therapeutic alternatives. The aim of this study is to evaluate the feasibility, safety, and efficacy of cell therapy based on mesenchymal stromal cells derived from adipose tissue intramuscular administration to patients with type 2 diabetes mellitus with critical limb ischemia and without possibility of revascularization.

**Methods:**

A multicenter, randomized double-blind, placebo-controlled trial has been designed. Ninety eligible patients will be randomly assigned at a ratio 1:1:1 to one of the following: control group (*n* = 30), low-cell dose treatment group (*n* = 30), and high-cell dose treatment group (*n* = 30). Treatment will be administered in a single-dose way and patients will be followed for 12 months. Primary outcome (safety) will be evaluated by measuring the rate of adverse events within the study period. Secondary outcomes (efficacy) will be measured by assessing clinical, analytical, and imaging-test parameters. Tertiary outcome (quality of life) will be evaluated with SF-12 and VascuQol-6 scales.

**Discussion:**

Chronic lower limb ischemia has limited therapeutic options and constitutes a public health problem in both developed and underdeveloped countries. Given that the current treatment is not established in daily clinical practice, it is essential to provide evidence-based data that allow taking a step forward in its clinical development. Also, the multidisciplinary coordination exercise needed to develop this clinical trial protocol will undoubtfully be useful to conduct academic clinical trials in the field of cell therapy in the near future.

**Trial registration:**

ClinicalTrials.govNCT04466007. Registered on January 07, 2020. All items from the World Health Organization Trial Registration Data Set are included within the body of the protocol.

## Background

Critical lower limb ischemia (CLI) is characterized by chronic pain at rest, ulcers or gangrene attributable to a proven occlusive arterial disease [1]. The evolution of CLI, in the context of a generalized atherosclerotic disease, implies high morbidity and mortality. CLI develops earlier and with greater intensity in patients with diabetes mellitus, with complications that may result in the amputation of the limb and even death [2, 3]. A public health goal of the standard of care is to decrease global cardiovascular risk through the control of risk factors by changes on lifestyle habits (quitting smoking, healthy diet, physical exercise) and pharmacological treatment (antiplatelet therapy, statins) [4]. However, there is insufficient evidence to show that this approach improves substantially the course of this disease [5]. Surgical or endovascular revascularization is often the treatment of choice, despite the fact that it is an invasive procedure that carries a high rate of complications. In addition, the health cost of this pathology is quite high. The treatment of the diabetic foot, and specifically critical limb ischemia, is the second most expensive complication in economic terms for the health system, just behind dialysis [6]. Efforts to prevent loss of the affected limb also include soft tissue debridement, minor amputations, and even skin grafts, all of which are costly procedures. In this context, given the high number of amputations that are still practiced annually worldwide, we are still in need of a cost-effective and easy-to-apply treatment [7]. Recently, research has shown that it is possible to improve tissue perfusion through therapeutic angiogenesis, making it one of the most promising strategies used to promote the proliferation of collateral vessels in ischemic tissues. In this sense, there are several methods to improve tissue perfusion, such as the administration of recombinant growth factors [8–11], or the constitutive expression of genes that encode for these factors through gene therapy [12]. Although both therapies have shown to promote angiogenesis in ischemic conditions in preclinical models [13], modest results were obtained when clinically applied [14–17]. In contrast, it has been shown both in animal models and in humans that cell therapy can induce therapeutic angiogenesis [18–20]. Cell-based therapies aiming to promote vascular regeneration gained interest with the discovery of a subpopulation of vasculogenic endothelial progenitor cells as reported by Asahara et al [17]. Human endothelial cell progenitors were isolated from peripheral blood by magnetic bead selection based on cell surface antigen expression. In animal models of ischemia, these cells have the ability to colonize the site of injury of ischemic tissue and secrete a number of vascular growth factors that can lead to clinically effective neovascularization [17]. It seems that these cells contribute to angiogenesis through the secretion of angiogenic cytokines and proteases such as matrix metalloproteases (MMPs), among others, helping the stability and growth of the endothelial and vascular network [21]. In 2002, the pioneering results of the TACT study (Therapeutic Angiogenesis using Cell Transplantation study) were published, consisting of a pilot uncontrolled safety study (*n* = 25) and a randomized controlled clinical trial to evaluate the use of mononuclear cells of bone marrow (BM) by intramuscular injection in 22 patients for treat peripheral arterial disease (PAD). Cell therapy with BM-derived mononuclear cells significantly improved ABI (difference 0.09 [95% CI 0.06–0.11]), transcutaneous oxygen pressure (pTcO2), (13 [9–17]), rest pain (− 0.85 [− 1.6 to − 0.12]) and pain-free walking time (1.2 [0.7–1.7]) in all treated patients [22]. Unfortunately, those with poorly controlled diabetes were excluded from the trial. As the prevalence of PAD increases in patients with type 2 diabetes mellitus (type 2 DM) it is logical to assume that this population group is a potential candidate for cell therapy [22]. Since then, several studies have been carried out in which the transplantation of the mononuclear fraction of BM or peripheral blood has been shown to improve the endothelial function in the territories in which they were administered [23] and to improve ischemic pain and the healing of ulcers [24]. In a phase I/II, open-label, non-comparative clinical trial, safety and feasibility of treatment with autologous mesenchymal stromal cells (MSC) derived from BM and administered intra-arterially in patients with type 2 DM and lower limb ischemia was studied. All of the participants (*n* = 20) showed an increase in leg vascularization demonstrated by angiography and 100% of the ulcers healed. During the first year of follow-up, 7 minor amputations were performed, but no patient suffered a major amputation [25]. Cañizo et al. reported the first case of a patient with CLI treated with peripheral blood CD133+ cells. There were no major amputations and after 17 months of follow-up, patients experienced symptomatic and functional improvement. The group also observed the appearance of blood flow in the posterior tibial artery that was absent before the procedure [26]. The search for the most appropriate cell type for this pathology remains a challenge today [27], but the use of MSC is gaining prominence. The fact that these cells have trophic, immunomodulatory, and anti-inflammatory properties and liposuction is an easy and minimally invasive technique places them as suitable candidates for clinical use. The study that we propose here focuses on the development and optimization of an advanced therapy medicinal product (ATMP) based on adipose tissue-derived MSC (Ad-MSC) to be administered intramuscularly to patients with type 2 DM and CLI within a multicenter randomized phase II clinical trial, favoring the translation of this cellular therapy to clinical practice. If proven safe and effective, an accessible, easy and minimally invasive treatment will be available for patients without any other option or that can even be associated with existing surgical treatments and improve their results.

The objectives of this study are the following:

1. To evaluate the safety and tolerability of the intramuscular administration of allogeneic Ad-MSC in patients with type 2 DM with critical lower limb ischemia and no possibility of revascularization
2. To evaluate the preliminary efficacy of the treatment
3. To evaluate the quality of life of participants of the study after treatment administration

## Methods

### Study design

This is a study protocol of a multicenter, randomized, placebo-controlled, double-blinded, dose-finding, phase II clinical trial of three parallel groups to evaluate safety and efficacy of the intramuscular administration of allogeneic Ad-MSC in diabetic patients with CLI and without possibility of revascularization, over conventional treatment. The overall study design is reported according to the CONSORT statement and agrees with the SPIRIT 2013 checklist.

**Fig. 1:**
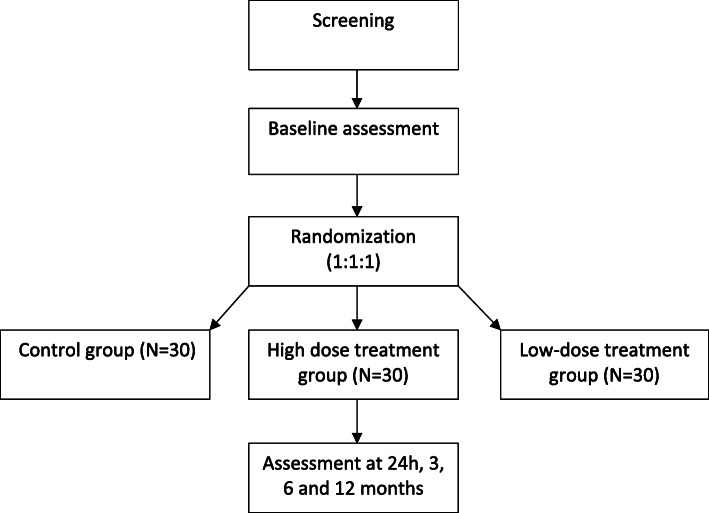
Flowchart: 90 patients will be recruited in the study and randomly allocated to three groups (high-dose group = 30; low-dose group = 30; control group = 30). The assessment will be done 24 h and 3, 6, and 12 months, respectively

A total of 90 eligible patients will be recruited from ten academic hospitals in Spain (Jimenez Diaz Foundation University Hospital, La Paz University Hospital, Navarra University Clinic, Gregorio Marañón General University Hospital, Virgen de la Arrixaca University Clinical Hospital, Salamanca University Hospital, Victoria Eugenia-Cruz Roja Española Hospital, La Fe Clinical and Polytechnic University Hospital, Valladolid Clinical University Hospital and Alicante General University Hospital). Participants will be randomly assigned into control group, low-cell dose treatment group, or high-cell dose treatment group, at a ratio 1:1:1.The flowchart of the trial is presented in Fig. 1 and the study procedures schedule is shown in Table 1. Recruitment period will last 1.5 years, and follow-up period will be 1 year. The expected total duration of the study, from the first visit of the first patient to the last visit of the last patient, will be 2.5 years.

**Table 1:** Schedule of study procedures

	Study period						
	Enrolment		Treatment	Follow-up			
Timepoint	***V-1, selection visit***	***V0, baseline visit***	V1, treatment	***V2, 24 h***	***V3, 3 m***	***V4, 6 m***	***V5, 12 m***
	***Day − 30 to day − 21***	***Day − 20 to − 1***	Day 0	***Day 1***	***Day + 90 ± 7***	***Day + 180 ± 15***	***Day + 365 ± 15***
***Eligibility criteria***	X	X					
***Informed consent***	X						
***Anamnesis***	X						
***Physical examination***	X		X	X	X	X	X
***Blood test***	X				X	X	X
***Urine pregnancy test***	X						
***Rutherford-Becker category***	X						
***Ankle-arm index***	X				X	X	X
***Ulcers evaluation (Wifi classification)***	X				X	X	X
***VAS scale***	X			X	X	X	X
***Gastrocnemius muscle perimeter***	X				X	X	X
***Temperature of the limb***	X				X	X	X
***Neuropathic symptoms***	X				X	X	X
***Quality of life scales (SF-12 and VascuQol-6)***	X				X	X	X
***MRI***	X						X
***Randomization***		X					
***Pre surgical evaluation***		X					
***Treatment administration***			X				
***Concomitant medication***	X	X	X		X	X	X
***Adverse events***			X	X	X	X	X

*MRI* magnetic resonance imaging, *h* hours, *m* month, *v* visit

### Recruitment of eligible participants

Patients will be recruited by the attending clinicians (vascular surgeons) among the population of chronic, type 2 DM patients with peripheral arteriopathy that is usually under periodic follow-up in the outpatient clinic. Proposal to participate in the clinical trial will take place in one of these routine outpatient visits. Clinicians will explain to each subject the nature of the study, its purposes, procedures, expected duration, and the potential risks and benefits related to participation in the trial, as well as any inconvenience that this may entail. Patients will have enough time to read and understand the explanations before dating and signing the informed consent and will receive a copy of the signed document. Eligible patients will be (1) those aged between 40 and 90 years; (2) with type 2 DM diagnosed for more than one year; (3) with severe vascular arteriosclerosis, defined as Rutherford-Becker (RB) category 4 and 5 [23], mono or bilateral; and (4) with impossibility of surgical or endovascular revascularization or failure of revascularization surgery performed, at least 30 days before inclusion in the study. Patients with CLI and tissue loss in the target limb (RB category 6) or previous major amputation in the target limb will be excluded from the trial. Detailed eligibility criteria are described in ClinicalTrials.gov (NCT04466007). During trial conduct, periodic contacts with investigators and a trial newsletter are foreseen to assess possible difficulties in recruitment in advance and to keep investigator team informed of recruitment evolution. No specific methods to minimize attrition are foreseen. Eligible patients are severely ill patients that are usually adherent to medical visits, and thus a high drop-out rate is not expected.

### Blinding, randomization and allocation concealment

Masking of participants will be guaranteed as follows: the solution for intramuscular infusion of Ad-MSC (active treatment) will have the same aspect as HypoThermosolFRS (placebo), and the syringes will be identified by a label that will exclusively contain the information corresponding to the clinical trial and the patient code. However, since the density of each product may be different, it is assumed that there is a risk that the clinical investigator administering the treatment may know which treatment is being applied. For that reason and to ensure double blind, each center will count, as a minimum, with two investigators per enrolled patient: a non-blinded investigator who will administrate cellular therapy and a blinded investigator who will perform the assessment of participants as described in Table 1. Randomization will be performed through the electronic case report form (eCRF). Study subjects will be assigned to group 1, 2, or 3, at a ratio 1:1:1, based in a random block sequence prepared by the sponsor’s statistical service. The assigned group information from each participant will not be visible in the eCRF. Then, an email with the assigned treatment group information of the participant will be sent automatically to non-blinded team. No stratification process has been designed in the randomization scheme. If a phase III clinical trial is subsequently developed, stratification will be defined, according to preidentified factors that could influence efficacy outcomes. During the study, masking can be broken in the event of a serious adverse event (SAE) related to the study medication that requires urgent medical treatment. The researcher will notify the sponsor within a maximum period of 24 h and sign the SAE notification form that will be sent by fax or email to the person in charge of pharmacovigilance of the study. The sponsor will communicate to the investigators any information that may affect the safety of the trial subjects as soon as possible. In the event of unmasking, blinding will be maintained for those responsible for evaluating the primary variable and for those responsible for data analysis and interpretation of the results.

### Interventions

**Fig. 2:**
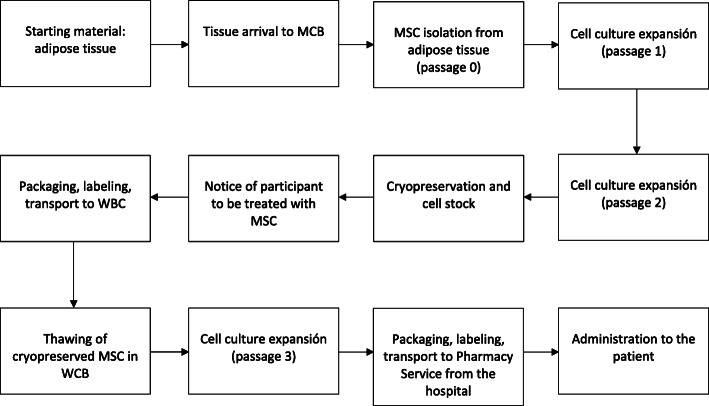
Cell therapy product flowchart. From GMP laboratory to the patient. MCB, master cell bank; WCB, working cell bank; MSC, mesenchymal stromal cells. Ad-MSC are isolated from the adipose tissue sample and cultured at two passages until reaching a minimum dose of 250 × 10^6^ cells. Characterization of the cells and quality controls are established during the whole process. When a study subject is assigned to active treatment group, the sponsor notifies the assigned WCB laboratory to thaw and culture the cells in one passage. The batch is then packaged, labeled, and sent to the pharmacy service of the intended hospital. Manufacture process has been authorized by the Spanish competent authority (AEMPS), PEI number 15-103 Version 4:7/07/2019.

**Fig. 3:**
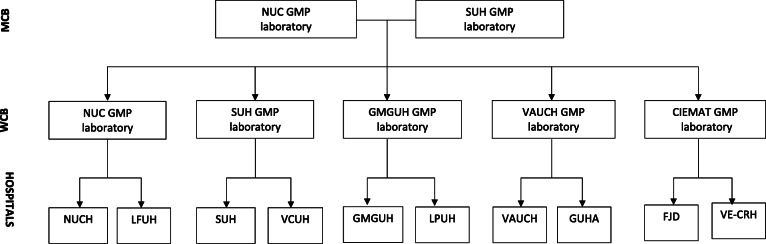
MCB and WCB laboratories. Different laboratories have been assigned to the hospitals. MCB, master cell bank; WCB, working cell bank; NUC, Navarra University Hospital; SUH, Salamanca University Hospital; GMGUH, Gregorio Marañon General University Hospital; VAUCH, Virgen de la Arrixaca University Clinical Hospital; LFUH, La Fe University Hospital; VCUH, Valladolid Clinical University Hospital; LPUH, La Paz University Hospital; VE-CRH, Queen Victoria Eugenia-Cruz Roja Hospital; FJD, Jimenez Diaz Foundation University Hospital; HGUA, General University Hospital Alicante

Ad-MSC will be obtained from young healthy donors who previously gave their informed consent. Good manufacture practice (GMP)-accredited cell therapy laboratories from University of Navarra Clinic and from Salamanca University Hospital will be responsible of the master cell bank (MCB). MCB is the system whereby successive batches of the cell therapy product are manufactured by isolation and expansion of cells derived from a single adipose tissue sample. In this way, we ensure stability and uniformity in the treatment. MCB laboratories will send batches of cryopreserved cells to working GMP cell bank (WCB) laboratories. When a participant of the study is assigned to active treatment group, the sponsor will notify the assigned WCB laboratory to thaw and culture the cells in one passage. Finally, the batch will be packaged, labeled, and sent to the pharmacy service of the corresponding hospital (see Figs. 2 and 3). Three parallel groups have been designated as follows:

- *Group 1*. Control group (*n* = 30). Placebo will consist of HypoThermosolFRS contained in an identical vial to that of the investigational medicinal product (IMP) and with the same volume.
- *Group 2*. Low-cell dose treatment group (*n* = 30). This group will receive a single intramuscular administration of 1 × 10^6^ cells/Kg weight.
- *Group 3*. High-cell dose treatment group (*n* = 30). This group will receive a single intramuscular administration of 2 × 10^6^ cells/Kg weight.

**Fig. 4:**
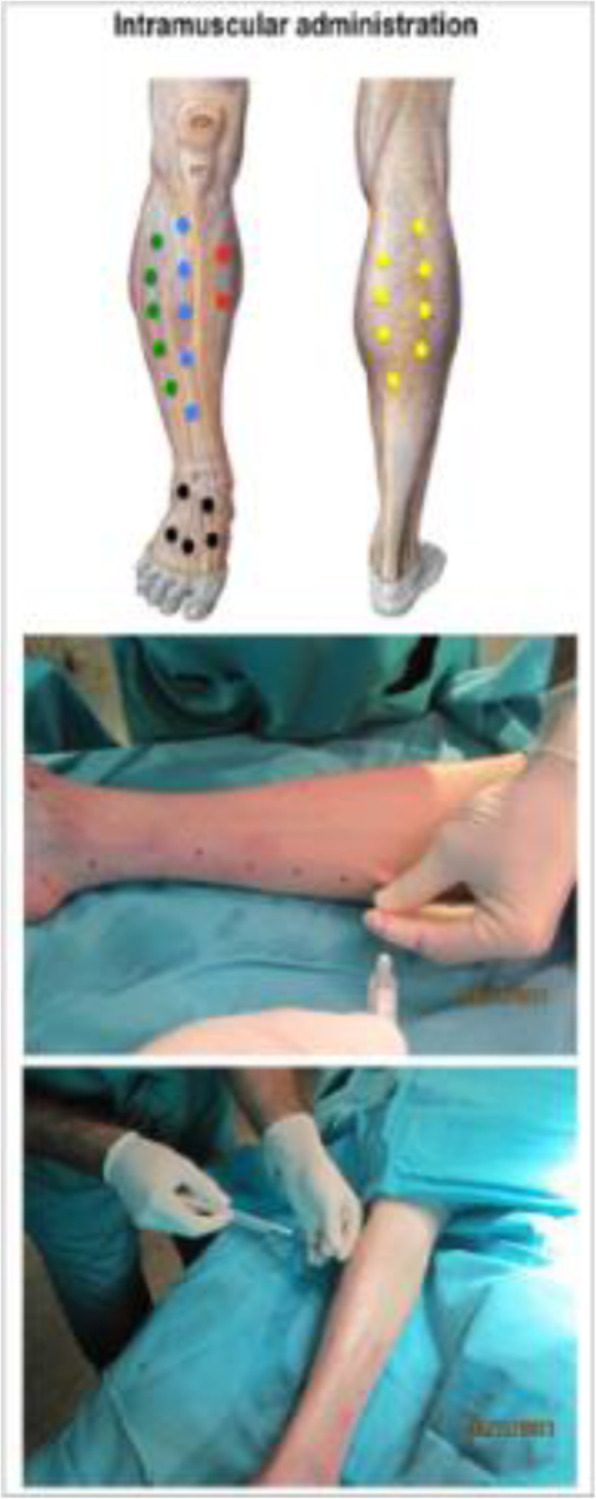
Once the targeted muscular injection points are selected (upper panel), repeated administrations of cell therapy are directly, easily, and minimally invasive administered into the lower part of the limb (lower panel)

Regarding the allogeneic treatment presented in this protocol and possible risk of immune rejection, MSC are considered to have an immunoprivileged status. These cells display a low expression of MHC-HLA class I and are constitutively negative for HLA class II [28], thus avoiding the presentation of antigens to cytolytic T lymphocytes and immunological rejection. Furthermore, an autologous use would entail MSC isolation from multipathological patients. It has been suggested that the hyperglycemic environment and metabolic disorders associated with diabetes affect the biological properties and angiogenic capacity of cells [29–31]. Ad-MSC isolated from young healthy donors are more likely to present uniform cellular properties [7]. Treatment will be administered intramuscularly at the infrapopliteal level, at 25 points in the ischemic area parallel to the vascularization of the affected limb (Fig. 4). Treatment administration will be performed in the operating room, after patients have gone through mandatory presurgical assessment. As the IMP consists of a living drug, the importance of standardizing handling of Ad-MSCs was emphasized during the design of the clinical trial. Hence, a protocol for the management and administration of the medication was established. Doses are proposed according to previous experience of the group and bibliographical data. In previous trials (Phase I/IIa), 1 × 10^6^ cells/kg of patient weight have been used [7, 22, 32]. In parallel and considering the results of these tests, as well as the safety demonstrated in the administration of Ad-MSC, it seems justified to check whether a higher cell dose could improve the results obtained to date. In case of bilateral affection, the limb to be treated will be the more damaged one. In addition to the IMP administration and study procedures, patients will be managed according to routine clinical practice. Patients may voluntarily discontinue the study and will be withdrawn from the study if they present any clinically relevant condition that may represent a risk. After the end of the trial, patients will be managed according to best clinical practice.

### Outcome measures

#### Primary outcome measure

Safety will be assessed comparing the rate of treatment related complications between groups (proportion of treatment related complications among groups) at 24 h and 3, 6, and 12 months from treatment administration, including those related with anesthetic procedure, IMP administration and those occurring after the procedure.

#### Secondary outcome measures

Efficacy will be assessed through the following variables:

(a) Vascularity in the target limb will be assessed through mean change in percentage of vascular flow measured by MRI in the target limb at 12 months from baseline between groups. MRI will be centrally read by a blinded radiologist. (b) Disease severity will be evaluated through percentage of patients with an improvement in RB category at 3, 6, and 12 months from baseline [33] between groups. This scale is usually used in patients with chronic arterial disease to classify chronic arterial disease into clinical categories (category 0, asymptomatic; category 1, mild claudication; category 2, moderate claudication; category 3, severe claudication; category 4, pain at rest; category 5, minor tissue loss; category 6, ulcer o gangrene). (c) Ulcer healing (if any) will be assessed through the percentage of patients with a decrease in SVS-“WIfI” wound classification [34, 35] at 3, 6, and 12 months from baseline between groups. SVS-WIfI classification aims to be as functional in diagnosing PAD as the tumor-node-metastasis-based diagnosis system (TNM). Three measurable parameters are collected: wound (extension and depth), ischemia (blood flow), and foot infection (presence and extension). (d) PAD evolution will be evaluated by % of patients with an increase in ankle-brachial index at 3, 6, and 12 months from baseline between groups [36]. (e) Pain intensity will be assessed through mean change in visual analogue scale (VAS) from 0 to 10 cm, as described by the patient (0, absence of pain; 1–3, mild; 4–7, moderate; 8–10, severe) and percentage of patients with an improvement in pain intensity at rest at 3, 6, and 12 months from baseline between groups. (f) Percentage of patients with a decrease in temperature (°C), increase in gastrocnemius muscle perimeter (cm) and decrease in neuropathic symptoms of the treated limb compared with contralateral at 3, 6, and 12 months from baseline. (g) Mean change in percentage of amputations in each group at the end of the study will be registered.

#### Tertiary outcome measures

Quality of life will be assessed through mean improvement scores obtained from the generic short-form-12 (SF-12) questionnaire [37] and peripheral arterial disease specific Vascular Quality of Life Questionnaire-6 (VascuQoL-6) [38] at 3, 6, and 12 months from baseline between groups. SF-12 scale is a reduced version of the SF-36 questionnaire, easy-to-apply to assess the functional capacity of people over 14 years of age. It includes the following dimensions: physical role, body pain, mental health, general health, vitality, social function, and emotional role. In this case, we will use version 2, in which 50 (with an SD of 10) is taken as the mean of the general population. Values above 50 should be interpreted as best, while values below 50 should be interpreted as worse than the mean. For each of the items in each dimension, the score ranges from 0 (the worst health for that dimension) to 100 (the best health for that dimension). VascuQoL-6 questionnaire consists of six questions with a score of 1–4 in each question. To obtain the general score of the questionnaire, it is necessary to add all the points obtained in each question. A high final value indicates a better state of health. As there is no validated version in Spanish, our research group is performing a cross-cultural adaptation and validation of this questionnaire in Spanish.

### Adverse events

An adverse event (AE), also referred to as an adverse experience, can be any unfavorable and unintended sign (i.e., an abnormal laboratory finding), symptom, or disease temporally associated with the use of a drug (or other investigational therapy) and does not imply any judgment about causality. An AE can arise with any use of the drug (i.e., off-label use, use in combination with another drug and with any route of administration, formulation, or dose, including an overdose). A suspected adverse reaction (SAR) means any AR for which there is a reasonable possibility that the drug caused the AE. An AE is considered “serious” if (SAE), in the view of either the principal investigator, if it results in any of the following outcomes: death of subject; event with risk to life, that is, there was an immediate risk of subject death at the time the reaction was observed; hospitalization or prolongation of hospitalization; persistent or significant disability/incapacity; congenital anomaly; or birth defect or any other significant medical condition, i.e., important AEs that do not pose an immediate risk to life and do not result in death or hospitalization but may endanger the subject or may require intervention to prevent one of the outcomes listed. A suspected adverse reaction is considered “unexpected” if it is not listed in the safety reference information for the IMP or is not listed at the specificity or severity that has been observed or is not consistent with previously available, known risk information.

Causality and severity assessment of any occurring AE will be performed by the principal investigator. AE will be registered from administration of IMP to end of study visit. Investigator will actively ask patients about AE occurrence in every study visit. Also, AE may be spontaneously reported by the patients. All of them will be appropriately registered in the patient medical records. Only AR and SAE will be registered in eCRF and reported according to legal requirements. For the purposes of this study, proportion of amputations in each study group is a secondary outcome and will be registered specifically, so notification as SAE will not be performed unless its clinical course is different of more severe than expected.

### Statistical considerations

#### Sample size calculation

A formal size calculation based on the expected differences for the primary outcome has not been performed. This is a phase II, dose-finding study, and no previous data on the preliminary efficacy of Ad-MSC with CLI including type 2 DM patients are available to date. A sample size of 30 patients per group was deemed appropriated based on the possibility to reach normality and the feasibility to recruit 90 eligible patients in 10 university hospitals.

#### Data collection and management

Relevant information for the study will be registered in electronic medical records and then entered in the eCRF in a pseudonymized fashion. All of these data will be also documented in the investigator’s file, which will be saved in key-locked cabinets. Monitor may need to verify the original data against the subject’s medical history and sponsor will have access to the final trial dataset.

#### Statistical analysis

Primary, secondary, and tertiary outcomes (safety, efficacy, and quality of life) will be assessed in intention to treat (ITT) and per protocol (PP) populations [39]. ITT population will include all patients who sign the informed consent and receive the investigational product. PP population will include patients who complete the 12-month follow-up period. A descriptive analysis of baseline characteristics of patients included in three groups will be performed. Quantitative variables will be expressed as mean and/or median and standard deviation and/or range. Qualitative variables will be expressed as percentages. When possible, the 95% confidence interval of each of the estimates made will be calculated. To analyze the primary outcomes, 24 h and 3, 6, and 12 months complication rates will be described and compared for the three groups using the Chi-square test or, if necessary, the Fisher exact test. The analyses of the secondary and tertiary outcomes will be performed as follows: qualitative variables will be described in terms of the percentage of improvement of the medical condition in each group and compared using the Chi-square test, or alternatively Fisher’s exact test. VAS scale, SF-12 scale, VascuQuol-6 scale, and the rest of the quantitative variables will be assessed by describing changes in variables at 3, 6, and 12 months from baseline, in an absolute and relative way. Changes at 3, 6, and 12 months in the three groups will be described and compared using the analysis of variance test, or the Kruskal-Wallis test, depending on whether or not the data follow a normal distribution. In all comparisons, a global comparison of the three groups and comparisons between groups by pairing will be carried out (low-dose treatment versus placebo, high-dose treatment versus placebo, high-dose treatment versus low dose, both treatment groups versus placebo). P values will be corrected by Bonferroni test. Regarding handling of missing data, a complete case analysis will be performed if percent of missing data is less than 5%. In another case, a data imputation will be performed for those variables with 25% or less missing values. Variables with more than 25% missing values will be removed from the analysis. The statistical analysis will be carried out by the statistical service of the Jiménez Díaz Foundation University Hospital. Data analyst will be blinded.

So far, no plans to use data generated in this trial for additional studies is foreseen. Nevertheless, and according to GDPR requirements, a statement has been included in the informed consent form informing the patient about this possibility and asking her/him for permission.

### Auditing

The study is supported by SCReN (Spanish Clinical Research Network) funded by ISCIII-General Subdivision for Evaluation and Promotion of Research, project PT17/0017/0022-PT20-00142 integrated in the 2013–2016 NationalI+D+iPlan, and co-financed by the European Regional Development Fund (FEDER). SCReN will be in charge of project management, monitoring, and pharmacovigilance activities.

## Discussion

Patients with type 2 DM tend to develop more advanced forms of CLI [40], displaying a combination of macro and microangiopathy, neuropathy with loss of sensation, tissue damage, increased risk of infection, endothelial inflammation, pro-thrombosis, and a greater inflammatory response, leading to a more distal, diffuse, and severe disease [41]. Currently, 6000 amputations are performed yearly in our country, and more than 70–80% are due to PAD. Despite its dramatic social impact, treatment options are limited due to the very negative long-term prognosis, with an increase in mortality after 10 years, 15 times higher than the patients without PAD [42].

Regenerative medicine is making possible to address unmet therapeutic needs. Specifically, cell-based therapy is positioning itself as one of the most promising approaches, generating great progress and a new frontier in healthcare. Cell therapy programs have opened on a wide range of fields with translational goals, providing sufficient evidence to show that it is a safe treatment [25–27, 43, 44]. Efficacy of the intramuscular administration of autologous Ad-MSC in type 2 DM patients with CLI was demonstrated in the pilot study conducted by Riera et al., showing a statistically significant improvement in health-related quality of life, an increase in ABI, and a decrease in RB category in the post-treatment period [32]. To our knowledge, no randomized clinical trial protocols consisting of the administration of Ad-MSC in type 2 DM patients with CLI have been published to date. Given that current treatment is not established in daily clinical practice, it is essential to promote and systematize the knowledge already obtained with the aim of developing well-designed cell therapy-based clinical trials. In our previous experience, management of the IMP to ensure the viability and survival of MSC is essential and was discussed in multiple meetings, leading to the development of a protocol for the correct handling and administration of the cell-based therapy. The implementation of this protocol implies a great coordination effort, in which basic and clinical researchers worked together to ensure that the living medicinal product reaches from the laboratory to the patient. Finally, the design of a multicenter independent-driven clinical trial based on cell therapy administration represents a challenge requiring a large logistical organization (as shown in Figs. 2 and 3) and great involvement and collaboration from the entire research team for the study to be feasible. “Feasibility” of the NOMA clinical trial also refers to the following characteristics of the proposed intervention: (i) On the one hand, it is an allogeneic product, in which 30 batches of cell therapy at a dose of 1 × 10^6^ cells/kg of weight, and 30 batches of cell therapy at a dose of 2 × 10^6^ cells/kg of body weight are obtained from a single liposuction. In autologous uses, patient needs to undergo liposuction in a first stage, and the administration of the cell therapy in a second stage. (ii) On the other hand, an intramuscular administration has been proposed, technically simpler than intravascular administration. Hence, an easy-to-apply cell therapy is proposed in this protocol, contributing to the feasibility of the intervention in a clinical setting. This will undoubtfully be useful to conduct academic clinical trials in the field of cell therapy in the near future.

## Data Availability

The full trial protocol and amendment history, and the anonymized participant-level dataset, will be made publicly available as supplementary material with the study results publication and in ClinicalTrials.gov and Spanish clinical trials (ReEC) registers after clinical study report has been sent to the National Competent Authority.

## Abbreviations

ABI: Ankle-brachial index
Ad-MSC: Adipose tissue-derived mesenchymal stromal cells
ATMP: Advanced therapy medicinal product
BM: Bone marrow
CLI: Critical limb ischemia
EC: Ethics committee
eCRF: Electronic case report form
GMP: Good manufacture practice
IMP: Investigational medicinal product
ITT: Intention to treat
MCB: Master cell bank
MSC: Mesenchymal stromal cells
MRI: Magnetic resonance imaging
NOMA: No more amputations
PAD: Peripheral arterial disease
PP: Per protocol
RB: Rutherford-Becker
SAE: Serious adverse event
SF: Short form
SVS-WIfI: Society for Vascular Surgery-Wound Ischemia and Foot Infection Classification System
TACT study: Therapeutic Angiogenesis using Cell Transplantation study
Type 2 DM: Type 2 diabetes mellitus
TNM: Tumor-node-metastasis-based classification system
VAS: Visual analogue scale
VascuQoL: Vascular Quality of Life Questionnaire
WCB: Working cell bank
